# Resistance mechanisms of immune checkpoint inhibition in lymphoma: Focusing on the tumor microenvironment

**DOI:** 10.3389/fphar.2023.1079924

**Published:** 2023-03-07

**Authors:** Chunlan Zhang, Leiming Wang, Caigang Xu, Heng Xu, Yu Wu

**Affiliations:** ^1^ Department of Hematology, West China Hospital, Sichuan University, Chengdu, China; ^2^ Shenzhen Bay Laboratory, Center for transnational medicine, Shenzhen, China; ^3^ State Key Laboratory of Biotherapy and Cancer Center, West China Hospital, Sichuan University, Chengdu, China; ^4^ Department of Laboratory Medicine, Research Center of Clinical Laboratory Medicine, West China Hospital, Sichuan University, Chengdu, China

**Keywords:** immune checkpoint inhibition, the tumor microenvironment, resistance mechanism, lymphoma, metabolites

## Abstract

Immune checkpoint inhibitors (ICIs) have revolutionized the therapeutic strategies of multiple types of malignancies including lymphoma. However, efficiency of ICIs varies dramatically among different lymphoma subtypes, and durable response can only be achieved in a minority of patients, thus requiring unveiling the underlying mechanisms of ICI resistance to optimize the individualized regimens and improve the treatment outcomes. Recently, accumulating evidence has identified potential prognostic factors for ICI therapy, including tumor mutation burden and tumor microenvironment (TME). Given the distinction between solid tumors and hematological malignancies in terms of TME, we here review the clinical updates of ICIs for lymphoma, and focus on the underlying mechanisms for resistance induced by TME, which play important roles in lymphoma and remarkably influence its sensitivity to ICIs. Particularly, we highlight the value of multiple cell populations (e.g., tumor infiltrating lymphocytes, M2 tumor-associated macrophages, and myeloid-derived suppressor cells) and metabolites (e.g., indoleamine 2, 3-dioxygenase and adenosine) in the TME as prognostic biomarkers for ICI response, and also underline additional potential targets in immunotherapy, such as EZH2, LAG-3, TIM-3, adenosine, and PI3Kδ/γ.

## 1 Introduction

Over the past decades, inhibition on immune brakes effects of cytotoxic T lymphocyte-associated protein 4 (CTLA-4) and the programmed cell death protein 1 (PD-1) by using immune checkpoint inhibitors (ICIs) have revolutionized the therapeutic strategies of cancer treatment, and become routine part of care for more than 20 different indications, including solid tumors and hematological malignancies (Schoenfeld and Hellmann, 2020). However, as increasing attention has been attracted, accumulating evidence suggests that durable response is only achieved by a small percentage of patients, while the majority of patients do not respond initially (primary resistance) or experienced disease progress afterward (acquired resistance) (Antonia et al., 2019; Fradet et al., 2019; Mok et al., 2019). Notably, in lymphoma, the efficiency of ICIs varies dramatically among different lymphoma subtypes (Hatic et al., 2021). In some subtypes, initial responsiveness to ICIs is remarkably high, whereas primary resistance predominates in others (Hatic et al., 2021). On the other hand, the primary responders may usually experience disease progression over time as acquired resistance develops (Chen et al., 2019). Generally, the overall long-term control rate with ICIs is disappointing for lymphoma.

After numerous investigations on the large individual differences among patients in terms of treatment outcomes of ICIs, a series of biomarkers have been identified particularly for solid tumors, including clinical features (e.g., age and gender), tumoral characteristics (e.g., tumor mutation burden and PD-L1 expression) (Yan et al., 2018; Havel et al., 2019). On the other hand, tumor microenvironment (TME) is a complex network around tumor cells, participating in multiple processes, including tumor pathogenesis, progression and metastasis (Liu et al., 2021). TME not only play instrumental effect on carcinogenesis, but can also contribute to the resistance mechanisms of immunotherapy according to the increasing evidence (Lei et al., 2020; Liu et al., 2021). Given that lymphoma cells originate and spread in lymphoid organs, where immune cells are produced and reside, and where anti-tumor immune responses are typically triggered (Curran et al., 2017), lymphoma exhibited different TME characteristics compared to solid cancer (Fowler et al., 2016). Here, we systematically review the efficiency of ICIs and the current knowledge of TME’s roles in ICI resistance in lymphoma, aiming to underline the potential specific TME components as potential biomarkers for ICI treatment.

## 2 Efficiency of ICIs in lymphoma

Both efficiency and toxicity of ICI vary among patients with lymphoma. However, host factors rather than the malignant cells/TME are the main dominant to ICI-related toxicity, such as HLA types (Hasan Ali et al., 2019; Zhou et al., 2021). Additionally, the incidence and distribution of ICI-specific immune related adverse events is approximately similar between solid tumor and hemato-oncology in general (Hradska et al., 2021). Therefore, we mainly focus on the varied efficiency of ICIs in lymphoma and its different subtypes, which exhibited diverse primary resistance and duration of response (DOR) (Table 1).

**TABLE 1:** The primary resistance rates and the medians of DOR of ICIs in lymphoma.

Lymphoma subtype	Therapy	Primary resistance rate	Median of DOR m)	References
r/r cHL	Pembrolizumab	28%–35%	16.5	Armand et al. (2016), Chen et al. (2017), Chen et al. (2019)
	Nivolumab	13%–42%	7.8–24.3	Ansell et al. (2015), Younes et al. (2016), Armand et al. (2018), Bekoz et al. (2020)
	Sintilimab	20%	NR	Shi et al. (2019)
	Tislelizumab	13%	NR	Song et al. (2020)
	Camrelizumab	24%	NR	Song et al. (2019)
	Avelumab	58%	6.9	Herrera et al. (2021)
Newly diagnosed cHL	Nivolumab	16%	NA	Ramchandren et al. (2019)
r/r PMBCL	Pembrolizumab	52%–55%	NR	Armand et al. (2019)
r/r DLBCL	Nivolumab	90%–96%	NR	Ansell et al. (2019)
r/r FL	Nivolumab	96%	10.9	Armand et al. (2021)
r/r CTCL	Pembrolizumab	62%	NR	Khodadoust et al. (2020)
r/r NK/T cell lymphoma	Pembrolizumab	0%–43%	4.1	Kwong et al. (2017), Li et al. (2018)
	Sintilimab	25%	4.1	Tao et al. (2021)
	Avelumab	62%	NA	Frigault et al. (2020)
r/r PTCL	Nivolumab	67%	3.6	Bennani et al. (2022)
	Geptanolimab	59.6%	11.4	Shi et al. (2021)
r/r mature T cell lymphoma	Pembrolizumab	67%	2.9	Barta et al. (2019)

### 2.1 B cell lymphoma

Anti–PD-1/PD-L1 antibodies are proven to be remarkably beneficial in Hodgkin lymphoma (HL). In phase I/II clinical trials with nivolumab and pembrolizumab (humanized immunoglobulin G4 monoclonal antibodies targeting PD-1) in patients with relapsed and refractory classic HL (r/r cHL), the overall response rates (ORRs) were between 58% and 87%, with 12%–45% of patients achieving complete remission (CR) (Ansell et al., 2015; Armand et al., 2016; Younes et al., 2016; Chen et al., 2017; Armand et al., 2018; Chen et al., 2019; Bekoz et al., 2020). The medians of progression-free survival (PFS) were 11–15 months. Base on a favorable safety profile and long-term benefits, both drugs are clinically approved for the treatment of r/r cHL by the US Food and Drug Administration (FDA). Other anti-PD-1 antibodies, including sintilimab, tislelizumab and camrelizumab, also showed impressive effectiveness in phase II clinical trials in patients with r/r cHL, with ORRs of 80.4%, 87.1%, and 76.0%, respectively (Shi et al., 2019; Song et al., 2019; Song et al., 2020; Wu et al., 2022). Similarly, patients also received clinical benefits from anti–PD-L1 inhibitor (e.g., Avelumab). In a phase I clinical trial of 31 patients with r/r cHL treated with avelumab, the ORR was 41.9%, with a CR rate of 19.4% (Herrera et al., 2021). In newly diagnosed cHL patients, promising results were also achieved using PD-1 blockade alone or in combination with chemotherapy (Ramchandren et al., 2019; Allen et al., 2021). However, in cHL, although the initial responsiveness to PD-1 blockade was remarkably high, during long-term follow-up, relapse of disease was emerged, and ongoing responses were only sustained in less than one-third of all responders (Armand et al., 2018; Chen et al., 2019). The medians of DOR were 6.9–24.3 months. The majority of responders suffered from acquired resistance.

High responsiveness to PD-1 inhibitors has also been observed in primary mediastinal large B cell lymphoma (PMBCL). The ORRs were between 45%–48% in relapsed/refractory (r/r) PMBCL patients treated with pembrolizumab (Armand et al., 2019). Pembrolizumab was subsequently approved for the treatment of r/r PMBCL patients by FDA. In Phase II CheckMate 436 Study, the combination of nivolumab and brentuximab vedotin for r/r PMBCL patients resulted in an ORR of 73%, with a 37% CR rate (Chen et al., 2019). Similarly, acquired resistance to PD-1 blockade was also observed during follow-up. Benefits of PD-1 inhibitors were documented for patients with other subtypes of B cell lymphomas, including four patients with r/r primary central nervous lymphoma, one patient with primary testicular lymphoma (Nayak et al., 2017), and three patients with mediastinal gray-zone lymphoma (Melani et al., 2017). Nevertheless, safety and efficacy of PD-1 inhibitors in these subtypes requires to be testified by clinical trials with large patient size.

In sharp contrast, the responsiveness to ICIs was modest in diffuse large B cell lymphoma (DLBCL), follicular lymphoma (FL), and chronic lymphocytic leukemia/small lymphocytic lymphoma (CLL/SLL). In a phase II study in 121 patients with r/r DLBCL, administration of nivolumab resulted in an objective response rate of 10% in the autologous stem cell transplantation (ASCT)-failed cohort, and 3% in the ASCT-ineligible cohort. Only three patients had durable response (Ansell et al., 2019). In another phase II study, deploying pembrolizumab as post-ASCT consolidation in patients with r/r DLBCL did not seem to increase therapeutic benefit (Frigault et al., 2020). In FL, the activity of PD-1 inhibitors is also very limited. According to the CheckMate 140 trial, a phase 2 study of nivolumab for relapsed/refractory FL, the objective response rate was 4% (4 of 92 patients) (Armand et al., 2021). As for CLL/SLL, benefit of PD-1 inhibitor was only observed in Richter transformation. In a phase II study designed to test the efficacy and safety of pembrolizumab in CLL, objective response was observed in 44% (4 out of 9) patients with Richter transformation, while no responder was found in 16 relapsed CLL patients (Ding et al., 2017).

### 2.2 T- and NK-cell lymphoma

PD-1 inhibitors also showed their benefits in treating cutaneous T cell lymphoma (CTCL). In a multicenter phase II trial of pembrolizumab in 24 patients with advanced r/r mycosis fungoides or Sézary syndrome, the ORR was 38%, including 2CRs. The median DOR was not reached, with a median response follow-up time of 58 weeks (Khodadoust et al., 2020).

In the case of peripheral T cell lymphoma (PTCL), as it comprises highly heterogeneous subtypes of lymphoma that originate from mature T/NK cells, the responses to ICIs vary among different subtypes. Anti–PD-1/PD-L1 antibodies were reported to be efficient by several studies for r/r NK/T cell lymphoma. ORR of 100% was reported by a study of seven patients administered with pembrolizumab, with five reached CR, remission of which lasted during a median follow-up of 6 months (Kwong et al., 2017). In another study of pembrolizumab in r/r NK/T cell lymphoma, an ORR of 57% (4 of 7) was documented (Li et al., 2018). In a phase 2 study of sintilimab in 28 patients, the objective response rate reached 75%, whereas the median DOR was only 4.1 months (Tao et al., 2021). In a phase 2 study evaluating avelumab in 21 patients, the ORR was 38%, with five CRs. However, the median PFS was only 2.7 months because of frequent early progression (Frigault et al., 2020). In general, in patients with r/r NK/T cell lymphoma, although the initial response rate to ICIs were comparatively high, only a small fraction of patients achieved durable response.

In other subtypes of PTCL, nivolumab and pembrolizumab is less efficient. Modest activity and cases of hyper-progression were reported by a phase II study of nivolumab for r/r PTCL (Bennani et al., 2022). The study enrolled 12 patients, 6 with angioimmunoblastic T cell lymphoma (AITL), 3 with PTCL-NOS, and one patient each with anaplastic lymphoma kinase negative anaplastic large cell lymphoma (ALK-ALCL), enteropathy associated T cell lymphoma and hepatosplenic gamma delta T cell lymphoma. The ORR was 33%, with 2 CRs in patients with ALCL-ALK negative and AITL, whereas the median DOR was short at 3.6 months. Astonishingly, except for one CR, the remaining 3 out of 4 patients with AITL suffered from hyperprogressive disease, defined as time-to-treatment failure of equal or less than 1 month of therapy with symptomatic progression. In another phase 2 study investigating nivolumab in adult T cell leukemia/lymphoma (ATLL), the first three patients showed hyperprogressive disease after the first dose and the study was subsequently discontinued (Ratner et al., 2018). Similarly, the result of another phase 2 study of pembrolizumab for r/r mature T cell lymphomas was also suboptimal. The study enrolled 17 patients, including seven with PTCL-NOS, four with follicular T cell lymphoma. The ORR was 33%, whereas the DOR was only 2.9 months. The trial was halted early for futility, as the PFS at 3 months was below 50% (Barta et al., 2019). Surprisingly, geptanolimab, another anti-PD-1 humanized monoclonal antibody, showed much better result in a phase II study in patients with r/r PTCL from 41centers in China (Shi et al., 2021). Of 102 patients enrolled, 41 had PTCL-NOS, 23 had NK/T cell lymphoma, 12 had ALK-ALCL, 7 had ALK + ALCL, and 19 had other PTCL subtypes. The ORR was 40.4%, while the median DOR was 11.4 months, significantly longer than the median DORs observed in studies of nivolumab and pembrolizumab. Interestingly, the study initially excluded AITL patients to avoid possibility of hyperprogressive disease. However, a central pathology review revealed that four patients had AITL, and all of them had disease control, including two PRs and two SDs. No hyperprogressive disease was observed. Therefore, the application of PD-1 blockade in AITL is controversial and merits further investigation. Differences in efficiency may partially be attributed to the heterogeneity of PTCL, and requires further validation in larger randomized trials in PTCL subtypes. Predictive biomarkers for better responders are urgently needed.

In summary, in lymphoma, durable response to ICIs can be achieve only in a limited proportion of patients. In some subtypes, such as DLBCL and FL, primary resistance to ICIs occurs in the vast majority of patients, while in some other subtypes, such as NK/T cell lymphoma, although the sensitivity is relatively high, the benefits of ICIs is remarkably hindered by acquired resistance. Unveiling the mechanisms of primary and acquired resistance is fundamentally important to improve the benefits of ICIs in multiple aspects, including the identification of predictive biomarkers of response, and development of the combination therapies that reverse the resistance mechanisms.

## 3 Resistance mechanisms to immune checkpoint inhibition

Similar with solid cancer, the responsiveness to anti-PD-1 therapies in lymphoma is also mainly determined by underlying biologic features of the lymphoma cell itself, such as overexpression of PD-1 ligands (i.e., PD-L1 encoded by *CD274*), as well as the composition of the TME (Kline et al., 2020). However, overexpression of PD-L1 in lymphoma is commonly attributed to copy number gain of 9p24.1 (contained *CD274*) (Xu-Monette et al., 2018), which is rarely seen in solid cancer (Rooney et al., 2015). On the other hand, Epstein-Barr virus (EBV) infection is also commonly observed in lymphoma subtypes, and patients with both high PD-L1 expression and EBV infection are sensitivity to PD-1 blockade (Ansell et al., 2015; Ansell, 2021). Given the detailed mechanisms of these two prognostic factors have been well-established and reviewed, we mainly focus on the influence of the TME on the treatment outcomes in this review.

**FIGURE 1:**
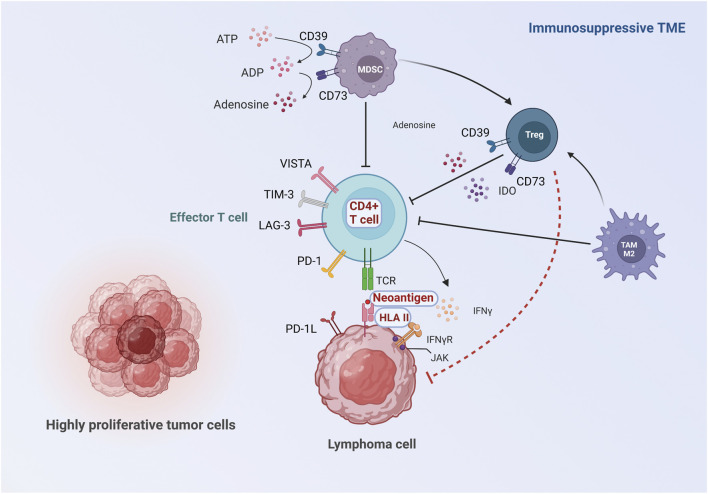
Proposed roles of TME in the mechanisms of ICI resistance. The immune response against lymphoma cells starts in the TME where neoantigens released by lymphoma cells are captured by antigen-presenting cells, followed by antigen presentation to T cells. Activated T cells kill lymphoma cells *via* IFN-γ signaling pathway. Inadequate activation and impaired function of effector T cells can be caused by insufficient neoantigen, aberrant HLA expression and defective IFN-γ signaling pathway. Moreover, the activation of effector T cells is inhibited by upregulation of other inhibitory checkpoint molecules, immunosuppressive metabolites and pro-tumorigenic cell populations in the TME. The major differences between lymphoma and solid cancer are highlighted: 1) The main effector T cells are CD4+T cells instead of CD8+T cells in some subtypes of lymphoma, and resistance to ICIs can be induced by aberrant expression of HLA II. Moreover, the characteristics of neoantigens in lymphoma are distinct from solid tumor. (Showed with red characters with white background). 2) Tregs may be anti-tumorigenic in some lymphoma subtypes, possibly due to the direct suppression of lymphoma cells (showed with red dash line).

Accumulating evidence implies that TME plays a fundamental role in immune surveillance, and contributes to the pathogenesis of malignancies (Liu et al., 2021). In solid cancer, resistance to ICIs is related to a non-inflamed TME, where effector T cells are physically excluded, or deprived of normal anti-tumor function *via* several kinds of mechanisms, including defects in neoantigen presentation, defective IFN-γ signaling pathway, upregulation of other inhibitory checkpoint molecules, and immunosuppressive metabolites and cell populations in the TME (Lei et al., 2020; Schoenfeld and Hellmann, 2020). Therefore, we here review the reported mechanisms of ICI-resistance in lymphoma and highlight their similarities and differences to those found in solid cancer, particularly the roles of the TME (Figure 1; Table 2).

**TABLE 2:** Comparation of lymphoma and solid cancer in resistance mechanisms to immune checkpoint inhibition.

Category	Solid cancer	Lymphoma	References
Inadequate T cell influx	High proliferative rate of tumor cells Other physical barriers against T cell priming	High proliferative rate of tumor cells	Fridman et al. (2012), Kline et al. (2020), Que et al. (2021)
Neoantigen presentation	More somatic mutations	Less somatic mutations Neoantigen provided by EBV infection	Alexandrov et al. (2013), Reichel et al. (2015), Kim et al. (2019)
Aberrant HLA expression on Effector T cells	Aberrant HLA class I expression on CD8^+^ T cells	Aberrant HLA class II expression on CD4^+^ T cells (cHL)	Liu et al. (2014), Carey et al. (2017), Nijland et al. (2017), Roemer et al. (2018), Liang et al. (2019)
Defective IFN-γ signaling pathway	mutations of *JAK1* or *JAK2*	No direct evidence yet	Lei et al. (2020), Schoenfeld and Hellmann (2020)
Upregulation of other inhibitory checkpoint molecules	LAG-3, TIM-3, VISTA, etc.	Similar	Aoki et al. (2020), Keane et al. (2020), Takata et al. (2020), El Halabi et al. (2021), Michot et al. (2021)
Immunosuppressive metabolites	IDO and adenosine	Similar	Ohta et al. (2006), Stagg et al. (2011), Masaki et al. (2018), Sugio et al. (2018), Wang et al. (2019), Kim et al. (2020b), Allard et al. (2022), Sun et al. (2022), Zhang et al. (2022)
Pro-tumorigenic cell populations	Treg	Anti-tumorigenic (cHL, FL, DLBCL)	Fowler et al. (2016), Peng et al. (2020), Maharaj et al. (2022)
		Pro-tumorigenic (CLL)	Maharaj et al. (2022)
	TAM	Similar	Armand et al. (2021), Gusak et al. (2021)
	MDSC	Similar	Liu et al. (2021)
	CAF	Anti-tumorigenic (DLBCL)	Kieffer et al. (2020), Liu et al. (2021)
		Pro-tumorigenic (FL)	

### 3.1 Inadequate T cell influx into the TME

The goal of ICI-based therapy is to reactivate the immune system and generate an effective antitumor immune response that targets lymphoma cells. Both CTLA-4 and PD-1 blockades reinvigorate the pre-existing antitumor T cell responses by removal of inhibitory signals (Wei et al., 2017). Similar to solid cancer, high level of tumor infiltrating lymphocytes (TILs) also predicts better response to anti-PD-1 therapy in lymphoma (Tumeh et al., 2014). The predict value of TILs is remarkable in B cell lymphoma, whereas the correlation of TILs level and sensitivity to ICIs is less preeminent in T- and NK-cell lymphoma, because of their heterogenous nature (Kline et al., 2020). In lymphoma, there are initially enough T cells in the TME because lymphoma cells originate from lymphoid organs where immune cells are produced. However, the innate high proliferative rate of tumor cells that can physically excludes the immune cells from entering the tumor core, and prevents an effective immune response (Fridman et al., 2012). For example, in high grade B cell lymphoma and Burkitt lymphoma, with strong cell autonomous growth and survival programs, tumor cells exhibit a high proliferative rate, which leads to aggressive clinical course and poor response to therapy (Kline et al., 2020; Que et al., 2021).

### 3.2 Inadequate T cell activation and impaired function

Given that lymphoma is a unique disease setting compared to solid cancer, as lymphoma cells originate from lymphoid organ, the mechanism underlying the dysfunction of effector T cells is different. It is suggested that tumor-specific T cells are primed in solid tumor, but become functionality compromised in the TME, whereas in lymphoma, tumor-specific T cells are never fully activated but are instead deleted or anergized upon initial antigen encounter (Curran et al., 2017).

The initial step of tumor-specific T cells activation is the proper presentation of neoantigens. Neoantigens are tumor-specific antigens derived mainly from genes mutated in tumor cells, and are targets of T cell mediated anti-tumor immune response. Neoantigens can also be produced by viral infection, alternative splicing and gene rearrangement (Wang et al., 2020; Zhang et al., 2021). Neoantigen depletion and defects in antigen presentation can both contribute to ICI resistance. Tumor mutational burden (TMB) is defined as the quantity of acquired mutations in the tumor genome. In solid tumor, high TMB can lead to a potential increase in the presentation of neoantigens, and result in an increase in the responsiveness to ICIs (Le et al., 2015; Le et al., 2017; Tang et al., 2019; Wu et al., 2020). In fact, a series of bioinformatic approaches have been developed to predict potential mutation-based neoantigens (O'Donnell et al., 2020; Reynisson et al., 2020; Hao et al., 2021), and successfully applied to clinical trials (Ott et al., 2017; Sahin et al., 2017; Ott et al., 2020; Ding et al., 2021), suggesting the crucial role of neoantigen in immunotherapy. Attempts have been made to stratify patients with lymphoma by TMB (Liang et al., 2019), categorized patients with HL into three subgroups according to TMB measured by comprehensive genomic profiling (Chen et al., 2021). A panel of 69 genes have been designed in DLBCL, and higher panel-TMB predicted inferior survival in patients treated with traditional chemotherapy. High TMB can be mostly caused by loss-of-function changes in DNA repair genes, and the inability to repair DNA mistakes is intimately tied to the instability of microsatellites (MSI). Therefore, MSI status is also a useful biomarker to predict clinical benefit from ICI therapy (Le et al., 2015), which has been studied in multiple lymphoma subtypes (Cuceu et al., 2018; Tian et al., 2020; Risinskaya et al., 2022). However, in the aforementioned studies in lymphoma, the clinical responses to ICIs in the stratified patients by TMB or MSI were not investigated.

Notably, compared to many solid tumors, next-generation sequencing revealed that lymphomas have a relatively small number of somatic mutations (Alexandrov et al., 2013). Particularly, the median number of somatic mutations observed in cHL is lower than many solid tumors (Reichel et al., 2015). In sharp contrast with this is the remarkably high sensitivity to PD-1 blockade in cHL, suggesting that the quality of neoantigen is as important as quantity in terms of responsiveness to ICIs in lymphoma. Additionally, EBV infection is closely related to multiple subtypes of lymphoma, providing an extra source of neoantigen and can improve the sensitivity to ICIs. For example, the response rate of pembrolizumab was higher in EBV-positive r/r non-Hodgkin lymphoma patients (Kim et al., 2019). In general, TMB and MSI might be useful biomarkers to screen patients with high sensitivity to ICIs, whereas TMB/MSI-based predictive system warrants further investigation in lymphoma. At the same time, identifying high quality neoantigens besides those provided by EBV infection is of the same importance.

The key step in T cell mediated immunity is the recognition of antigens on human leukocyte antigen (HLA) molecules of antigen-presenting cells. In solid tumors, ICIs eliminate tumor cells mainly by means of reactivating CD8^+^ cytotoxic T cells, *via* the presentation of antigen by HLA class I (Tumeh et al., 2014; Im et al., 2016). B2-microglobulin (β2M) is essential in stabilizing cell surface expression of HLA class I. Studies suggested that genomic alterations in *B2M* were related to both primary and acquired resistance to ICIs in some solid tumors, including melanoma and lung cancer (Gettinger et al., 2017; Sade-Feldman et al., 2017; Betof Warner et al., 2019; Lei et al., 2020). However, in cHL, it is suggested that the main effector cell of ICIs is CD4^+^ T cell instead of CD8^+^ cytotoxic T cell. First of all, the TME around Hodgkin Reed-Sternberg (HRS) cells is dominated by CD4^+^ T cells, and PD-L1^+^ HRS cells are more likely to be in direct contact with PD-1^+^CD4^+^ T cells than PD-1^+^CD8^+^ T cells (Carey et al., 2017). Moreover, lymphomas exhibit abnormally high *B2M* mutation rates compared with solid cancer, and about half of the patients carrying B2M aberrations show bi-allelic inactivating alterations (de Charette and Houot, 2018). Particularly, in contrast with the high responsiveness of anti-PD-1 therapy in patients with cHL, the HLA class I cell surface expression is lost in up to 70% of cHL cases (Oudejans et al., 1996; Liu et al., 2014; Nijland et al., 2017; Liang et al., 2019). Furthermore, expression of HLA class II but not HLA class I in HRS cells was predictive for CR or PFS after nivolumab therapy (Roemer et al., 2018). Patients with HLA class II–negative HRS cells were highly likely to experience primary resistance to ICIs. Notably, a subset of patients with MHC class II–negative HRS cells were responsive to nivolumab, but the DORs were rather short, indicating that the loss of HLA class II expression was also related to acquired resistance to ICIs, which appears in around 40% of cHL cases (Liu et al., 2014). The inactivation of the major histocompatibility complex class II transactivator (CIITA) is the common cause of aberrant HLA class II expression (Steidl et al., 2011). In addition, in HLA class II positive cHL cases, HLA-DM expression is lost in around a half of patients, leading to a functional loss of HLA class II, as HLA-DM is required to displace the class II invariant chain peptide and allow antigen loading into HLA class II (Nijland et al., 2017). In summary, in cHL, the mechanism of antitumor immunity depends on HLA class II–mediated antigen presentation to CD4^+^ T cells. Resistance to ICIs is closely relevant to dysfunction of HLA class II. Interestingly, unlike solid cancer cells, B cells can present antigens in the context of HLA class II molecules, recurrent HLA class II loss or downregulation may be an important mechanism of immune escape in B cell lymphomas (Curran et al., 2017).

The main effector cell in other lymphoma subtypes is less clear. PMBCL shares biological and clinical features with the nodular sclerosis variant of cHL (Rosenwald et al., 2003; Giulino-Roth, 2018), and also exhibited common loss of HLA class expression (Steidl et al., 2011). Multiple recurrent alterations have been reported, including *B2M* mutations, focal copy number losses of *B2M* and the *MHCI/MHCII* loci, and structure variants of *CIITA* and *EZH2* (Chapuy et al., 2019). *EZH2* mutation is linked to both loss of HLA class I and HLA class II expression, and a reduced TIL in TME (Ennishi et al., 2019). In some other lymphoma subtypes, loss of HLA expression was also common, including 62% of DLBCL, 77% of PCNSL and 87% of testicular lymphoma cases (Nijland et al., 2017). In DLBCL, the most frequent aberrant HLA expression is the loss of both HLA class I and HLA class II (35%). The underlying genomic alterations including *B2M* mutations/deletions, chromosome 6p21.32deletions, *CIITA* alterations and *EZH2* mutations (Riemersma et al., 2000; Challa-Malladi et al., 2011; Steidl et al., 2011; Ennishi et al., 2019). Recently, EZH2 appears to be a novel target for cancer treatment. In 2020, EZH2 inhibitors was approved by the FDA as a third-line option in r/r FL with EZH2 mutation (Cahill and Smith, 2022). Moreover, as EZH2 inhibitors also has activity in patients without an EZH2 gene mutation, it is also used in r/r FL without other treatment options (Cahill and Smith, 2022). EZH2 orchestrates the regulation of the innate and adaptive immune systems of the TME (Kim et al., 2020a). EZH2 inhibitors can significantly restore HLA expression in *EZH2*-mutated human DLBCL cell lines, suggesting that complementary therapeutic approaches combining ICIs with epigenetic reprogramming may reverse the resistance to ICIs (Ennishi et al., 2019). Clinical trials combining EZH2 inhibitors and ICIs are emerging. Disappointingly, according to the result of a phase Ib study, the combination of PD-L1 inhibitor and EZH2 inhibitor has modest anti-tumor activity in r/r DLBCL (Palomba et al., 2022).

The final step of effector T cells induced tumor cell death is *via* releasing proinflammatory cytokines, such as interferon-γ (IFN-γ) (Müller-Hermelink et al., 2008). IFN-γ triggers a signaling cascade in tumor cells *via* the JAK-STAT pathway that mediates both MHC class I and PD-L1 expression (Bach et al., 1997). In solid tumor, inactivating mutations of *JAK1* or *JAK2* contributed to both primary and acquired resistance to ICIs (Lei et al., 2020; Schoenfeld and Hellmann, 2020). Similarly, in a murine model of B cell lymphoma, the therapeutic effect of both PD-1 and CTLA-4 blockade was fully abrogated after ablation of IFN-γ (Ahmetlic et al., 2021). However, the direct evidence of the relationship of defective IFN-γ signaling pathway and ICI-resistance in lymphoma await to be addressed. Interestingly, in cHL and PMBCL, the recurrent copy gains of chromosome 9p24.1, resulting in co-amplification of *JAK2* and *CD274* (Green et al., 2010). Therefore, it is possible that the enhanced JAK2 activity also contributes to the high sensitivity of cHL and PMBCL to anti-PD-1 therapy.

### 3.3 Upregulation of other inhibitory checkpoint molecules in the TME

In solid tumor, upregulation of other immune checkpoint molecules in the TME have been documented, including lymphocyte activation gene-3 (LAG-3) (Gettinger et al., 2017), T cell immunoglobulin and mucin-domain containing-3 (TIM-3) (Koyama et al., 2016) and V-domain immunoglobulin suppressor of T cell activation (VISTA) (Kakavand et al., 2017). Elevated level of other immune checkpoint molecules is closely associated with resistance to PD-1 blockade (Koyama et al., 2016; Johnson et al., 2018). Several preclinical and clinical studies have demonstrated that the combination of LAG-3 or TIM-3 and PD-1 blockade overcome drug resistance and achieved good efficacy in solid tumor (Tian and Li, 2021; Wei and Li, 2022).

Similarly, the expression of LAG-3 and TIM-3 has been reported in multiple lymphoma subtypes. For example, in HL, LAG-3 is frequently found in the immune cells in TME, mainly CD4^+^ T cells, and appears to be higher in regions adjacent to HRS cells (El Halabi et al., 2021), whereas the expression of TIM3 is relatively low (Duffield et al., 2017). In PMBCL, high expression of LAG-3 in TME has also been reported, and the majority of LAG-3 positive cells are CD8^+^ T cells (Takata et al., 2020). In DLBCL, LAG3 expression have been observed on multiple immune cell types in TME, with highest expression on CD4^+^ regulatory T cells (Keane et al., 2020), and the expression of TIM-3 in immune T cells is also increased compared with healthy controls (Xiao et al., 2014). Moreover, expression of LAG-3 and TIM3 is remarkably elevated in NK/T cell lymphoma (Feng et al., 2018).

The role of LAG-3 in resistance to PD-1 blockade has been indicated by several studies in HL. First of all, overexpression of LAG-3 was observed on CD4^+^ T lymphocytes in TME after exposing to anti-PD-1 therapy (Michot et al., 2021). Moreover, using single cell RNA sequencing, a cluster of type 1 T regulatory (Treg) cells was identified to highly express LAG-3 but not PD-1, exhibiting a significant immunosuppressive effect on T cell (Aoki et al., 2020). Interestingly, this group of cells appeared to be spatially located close to HRS tumor cells with loss of MHC-II that can escape from the anti-tumor function of CD4^+^ T cells. In summary, similar to solid cancer, in lymphoma, upregulation of other immune checkpoint molecules may be related to drug resistance to PD-1 blockade *via* expressing on different T cell populations in TME. Currently, clinical trials evaluating the safety and efficacy profiles of anti-LAG-3 and anti-TIM-3 therapy in combination with PD-1 blockade in lymphoma is ongoing, such as NCT03311412 and NCT02061761, and results are eagerly awaited (Wei and Li, 2022).

### 3.4 Immunosuppressive and pro-tumorigenic cell populations in the TME

Besides the alterations in effector T cells, multiple TME cell types are involved in checkpoint blockade as immunosuppressor or pro-tumorigenic factors. As a subpopulation of CD4^+^ T cells, Tregs have immunosuppressive properties and depend on constitutive expression of FOXP3 transcription factor (Fontenot et al., 2005). In solid cancer, increased Treg cell frequency in the TME often correlates with poorer prognosis (deLeeuw et al., 2012). Higher density of Treg in the TME also predicts non-responsiveness to anti-PD-1/PD-L1 therapy in several types of solid tumor (Wu et al., 2018; Kumagai et al., 2020). However, the role of Tregs is complicated in lymphoma. Increased number of FOXP3^+^ Tregs is associated with superior outcome in cHL (Fowler et al., 2016). In general, higher FOXP3^+^ Treg level is associated with better outcomes in FL and DLBCL (Maharaj et al., 2022), but is controversial with conflicting evidence (Farinha et al., 2010; Cioroianu et al., 2019; Maharaj et al., 2022). Therefore, a meta-analysis including a total of 2,269 patients of various subtypes of lymphoma were conducted, revealing a significantly positive association of Treg with prolonged OS and PFS (Peng et al., 2020). However, in CLL, increased Tregs was found to be an adverse prognostic factor (Maharaj et al., 2022). Taken together, these studies indicate Tregs may play diverse role in different subtypes of lymphoma compared with solid tumor. The underlying mechanism is not clear, and it is possible that Tregs may directly suppress lymphoma cells (Fowler et al., 2016). Moreover, the predictive value of Tregs in lymphoma with anti-PD-1/PD-L1 therapy is unclear, which has been investigated with a few studies. For example, in a study investigating B-NHL patients treated with rituximab/ipilimumab (an anti-CTLA-4 antibody), CD45RA^−^ Treg to Treg ratio was elevated in responders compared to non-responders at baseline and following therapy (Tuscano et al., 2019).

Additionally, multiple TME cell types exhibit pro-tumorigenic characteristics, and actively participate in carcinogenesis, including M2 tumor-associated macrophages (TAMs), myeloid-derived suppressor cells (MDSCs), and N2 tumor-associated neutrophils (TANs). These cells are correlated with poor prognosis in various subtypes of lymphoma (Liu et al., 2021). In solid cancer, the presence of these pro-tumorigenic cell populations can provide an immunosuppressive environment to facilitate the resistance of cancer cells to ICIs treatment (O'Donnell et al., 2019; Genova et al., 2021). During tumorigenesis, TAMs transformed from the anti-tumorigenic M1 phenotype to the pro-tumorigenic M2 phenotype, which contributed to T cell dysfunction and exhaustion through secretion of cytokines and metabolites, and increased PD-L1 expression in tumor cells and other immunosuppressive cells (Mantovani et al., 2017; Dong et al., 2021; Pu and Ji, 2022). In multiple types of solid tumors, high degree of M2 macrophage infiltration in the TME were correlated to drug resistance of PD-1 blockade (Toulmonde et al., 2018; Kim et al., 2020a; Zhu et al., 2020). As a possible mechanism demonstrated in mouse model, PD-1 monoclonal antibodies (mAbs) were captured within minutes from the T cell surface by PD-1 negative TAM *via* interaction between FcγR on the surface of TAM and Fc domain of the PD-1 mAbs. Blockade of FcγRs prior to PD-1 mAb administration remarkably prolonged the binding of PD-1 mAb to CD8^+^ T cells and promoted tumor regression (Arlauckas et al., 2017). Similarly, a lower M2 macrophage level correlated with higher PFS and CR during nivolumab treatment in cHL (Gusak et al., 2021), while high expression of a set of TAM genes was associated with reduced PFS in FL (Armand et al., 2021). Therefore, the level of M2 macrophage and related gene signature may also serve as a potential predictive biomarker of responsiveness in lymphoma.

Cancer-associated fibroblasts (CAFs) are also pro-tumorigenic in solid tumor, and contain heterogenous subsets with distinct markers (Kieffer et al., 2020). Via single-cell sequencing analysis, correlation of some CAF subsets with resistance to PD-1 blockade was revealed in multiple cancer types, such as urothelial carcinoma (Luo et al., 2022). However, the roles of CAFs in lymphoma are ambiguous. For example, CAFs were recognized to be pro-tumorigenic in FL and related with inferior clinical outcome, whereas they were associated with favorable prognosis in DLBCL (Liu et al., 2021). The difference may be related to heterogenous nature of CAFs, and further investigation of subsets is required. Recently, singled-cell sequencing has also been utilized to characterize CAFs in lymphoma, identifying a novel subgroup of CAFs characterized by high expression of *EGR* genes, which facilitates T and NK cell expansion *via* epidermal growth factor receptor (Joo et al., 2022). In summary, the predictive value of CAFs in ICIs treatments remains to be elucidated in lymphoma.

Recent studies have verified that targeting pro-tumorigenic cell populations can efficiently improve the efficacy of ICIs (DeNardo and Ruffell, 2019). In fact, a series of phase I/II clinical trials targeting various types of pro-tumorigenic TME cells is currently ongoing in lymphoma (Liu et al., 2021). For example, tenalisib, by targeting PI3Kδ/γ, can significantly inhibit MDSCs and repolarize TAMs into M1 phenotype, and consequently lead to tumor regression (Locatelli et al., 2019). In a phase I/Ib study in patients with r/r T cell lymphoma, the ORR was 45.7% and median DOR was 4.9 months (Huen et al., 2020). In another phase I study of tenalisib, 35 patients with varied types of r/r hematologic malignancies including B cell and T cell lymphoma were enrolled. The ORR was 19% and the disease-control rate was 61%, with a median DOR of 5.7 months (Carlo-Stella et al., 2020). However, the phase I/II study, NCT03471351, assessing the combination of tenalisib and pembrolizumab in r/r cHL was terminated early, and the data was not published. More clinical trials are required to evaluate the safety and efficacy profiles of combination therapies of ICIs and agents targeting pro-tumorigenic cells in lymphoma.

### 3.5 Immunosuppressive metabolites in the TME

Besides the cell-cell interaction between TME and malignant cells, particular metabolites are also involved in inducing ICI resistance, and closely linked to aforementioned immunosuppressive cell populations. For example, indoleamine 2, 3-dioxygenase (IDO) can be expressed by TAMs. Tryptophan, which plays a fundamental role in cancer innate and adaptive immune tolerance, is a substrate of IDO *via* the kynurenine degradation pathway (Mbongue et al., 2015). Thus, IDO can induce a highly tolerogenic TME characterized by reduced T effector lymphocytes and NK cells, and an increased number of functionally active Treg cells and MDSCs (Ricciuti et al., 2019). The upregulation of IDO is involved in primary resistance to anti-CTLA-4 and anti-PD-1 therapy in solid tumor (Holmgaard et al., 2013; Botticelli et al., 2018; Brown et al., 2018; Kocher et al., 2021). IDO inhibitors had limited activity on their own but greatly enhanced the function of PD-1 blockade in phase I/II clinical trials (Prendergast et al., 2018). However, surprisingly, the combination of IDO inhibitors and PD-1 blockade failed in phase III trials with no benefit but increased adverse drug reaction events (Yan et al., 2018; Long et al., 2019). Nevertheless, recent promising reports demonstrated a remarkable efficacy of a immune-modulatory vaccine against IDO/PD-L1 combined with nivolumab in metastatic melanoma in a phase I/II trial (Kjeldsen et al., 2021). Further validation in larger randomized trials is required to confirm the clinical potential of this immunomodulating approach. Similarly, immune suppressive effect of IDO has also been documented in lymphoma. According to the recent study, 80% of mature T/NK cell neoplasms and around 30% of mature B cell lymphomas were IDO positive in immunohistochemistry (Kim et al., 2020a), and upregulated IDO expression in DLBCL was associated with a poor prognosis (Sun et al., 2022). In addition, inhibition of IDO suppressed DLBCL cell proliferation *in vitro* and impeded xenograft tumorigenesis *in vivo* (Sun et al., 2022). In HL, IDO was produced by macrophages and dendritic cells (DCs), and high IDO level was related to shorter OS (Masaki et al., 2018). In PTCL-NOS, high level of IDO was also found in tumor-infiltrating macrophages in a subgroup of patients with poor prognosis (Sugio et al., 2018). These studies indicated that IDO level influenced the outcome in multiple lymphoma subtypes, and IDO inhibitor might be beneficial. Taken together, it should be interesting to further investigate the contribution of IDO in ICI resistance in lymphoma. However, the unsatisfactory efficiency of IDO inhibitor plus anti-PD-1 therapy in solid tumor suggest that the addition of IDO inhibitor alone may be inadequate to reverse the mechanism of ICI resistance.

Another well reported immunosuppressive metabolite is extracellular adenosine, levels of which are high in hypoxic TME, but low in normal microenvironments (Leone et al., 2015). The ectonucleotidases (e.g., CD39 and CD73) can be expressed by MDSCs, and is capable to catabolize ATP to adenosine, and promote intracellular signaling through G protein-coupled receptors (GPCR), such as A2AAR and A2BAR (Augustin et al., 2022). The adenosine GPCRs can be found on all cell components of TME, including multiple types of immune cells, stromal cells and endothelial cells, with over-arching signaling effects leading to immune tolerance and malignant proliferation (Allard et al., 2020; Augustin et al., 2022). In addition, NAD^+^ shares structural features with ATP, and can also be metabolized to adenosine *via* an alternative pathway involving CD38, CD203a and CD73 (Horenstein et al., 2013). In fact, abnormality in adenosine metabolism that induces increased level of adenosine, is an alternative mechanism to explain ICI resistance in solid tumor. For example, CD73 expression suppressed lymphocyte functions, and increased in subsets of patients with melanoma progressing under anti-PD-1 therapy (Reinhardt et al., 2017; Turiello et al., 2022). In preclinical models of different solid tumors, the resistance to anti-PD-1/PD-L1 antibody was mediated by the upregulation of CD38 by the induction of both all-trans retinoid acid and IFN-β (Chen et al., 2018). CD38 expression on immune cells, especially macrophages, predicted response to ICIs in hepatocellular carcinoma (Panda et al., 2020), and combination of ICIs with anti-CD39 reverse the drug resistance to PD-1 blockade in a series of T cell poorly infiltrated tumor models (Li et al., 2019). In addition, blocking adenosine generation or signaling *via* CD73 or A2AAR, can also increase sensitivity of cancer cells to anti-PD-1 therapies (Vijayan et al., 2017). Currently, several clinical programs directed at A2 adenosine receptor (A2AAR and A2BAR), CD73 and CD39 are in development, and some clinical benefit was noted in solid cancer (Augustin et al., 2022; Chiappori et al., 2022). Similarly, adenosine pathway is also closely related to immune evasion in lymphoma. For instance in DLBCL, the numbers of CD8^+^ T cells with PD-1 and A2AAR expression were positively correlated with the number of dysfunctional CD8^+^ T cell, and worse clinical outcome (Zhang et al., 2022). Consistently, patients with CD73^+^ on tumor cells as well as A2AAR^+^ on tumor-infiltrating lymphocytes exhibited inferior survival in DLBCL (Wang et al., 2019). Moreover, pre-clinical studies have proved the anti-tumor effect of blocking adenosine pathway. For example, knocking-out A2AAR could significantly decrease tumor growth in a T cell lymphoma mouse model (Ohta et al., 2006), whereas CD73-deficient mice had increased antitumor immunity against inoculated lymphoma cells compared with wild-type mice (Stagg et al., 2011). More recently, CD73 deficiency was found to significantly delay CLL progression and prolonged survival in Eµ-TCL1 transgenic mice, and was associated with increased accumulation of IFN-γ^+^ T cells and effector-memory CD8^+^ T cells (Allard et al., 2022). Furthermore, adenosine pathway is also involved in drug resistance to immunotherapy for lymphoma. According to the recent study, adenosine critically impeded the therapeutic efficacy of anti-CD20 monoclonal antibodies against B cell lymphoma by impairing antibody-mediated cellular phagocytosis by macrophages and limiting the generation of anti-lymphoma CD8^+^ T cells (Nakamura et al., 2020). Based on these studies, it is reasonable to deduce that adenosine pathway might reduce the efficiency of ICIs in lymphoma in a manner similar to that in solid cancer. Currently, in lymphoma, the study concerning the combination of inhibitors targeting adenosine and ICIs in pre-clinical or clinical settings is scarce, and further investigation is in urgent need.

## 4 Conclusion and future perspectives

In lymphoma, the benefit of ICIs is significantly weakened by primary or acquired drug resistance. Based on current findings, TME may participate in ICI resistance by various mechanisms. Some of these mechanisms are similar to those found in solid tumors, while others differ. Efforts are still required to develop a deeper understanding of these resistance mechanisms, and translate current knowledge to clinical applications. Combination of therapies that reverse these resistance mechanisms may significantly improve the efficacy the ICIs. Based on current knowledge, EZH2, LAG-3, TIM-3, adenosine, and PI3Kδ/γ are additional potential targets in immunotherapy. The safety and efficiency of combinations of these targets with ICIs should be further testified in future studies. Moreover, several biomarkers are highlighted for better prediction of responders to ICIs. A predictive system for treatment response to ICIs should be constructed in future studies, and multiple measurements should be included, such as TILs levels, TMB and MSI, EBV infection, copy number gain of 9p24.1, expression of HLA, levels of other immune checkpoint molecules, and levels of immunosuppressive cell populations and metabolites in the TME.
